# Supplementary material for: DNA methylation-based classification of sinonasal tumors
Source: Nat Commun. 2022 Nov 28;13:7148. doi: 10.1038/s41467-022-34815-3 (PMC9705411; doi:10.1038/s41467-022-34815-3)
Supplement: Supplementary file 1 — Supplementary Information [file 41467_2022_34815_MOESM1_ESM.pdf]

Supplementary Information

Jurmeister et al., DNA methylation-based classification of sinonasal tumors

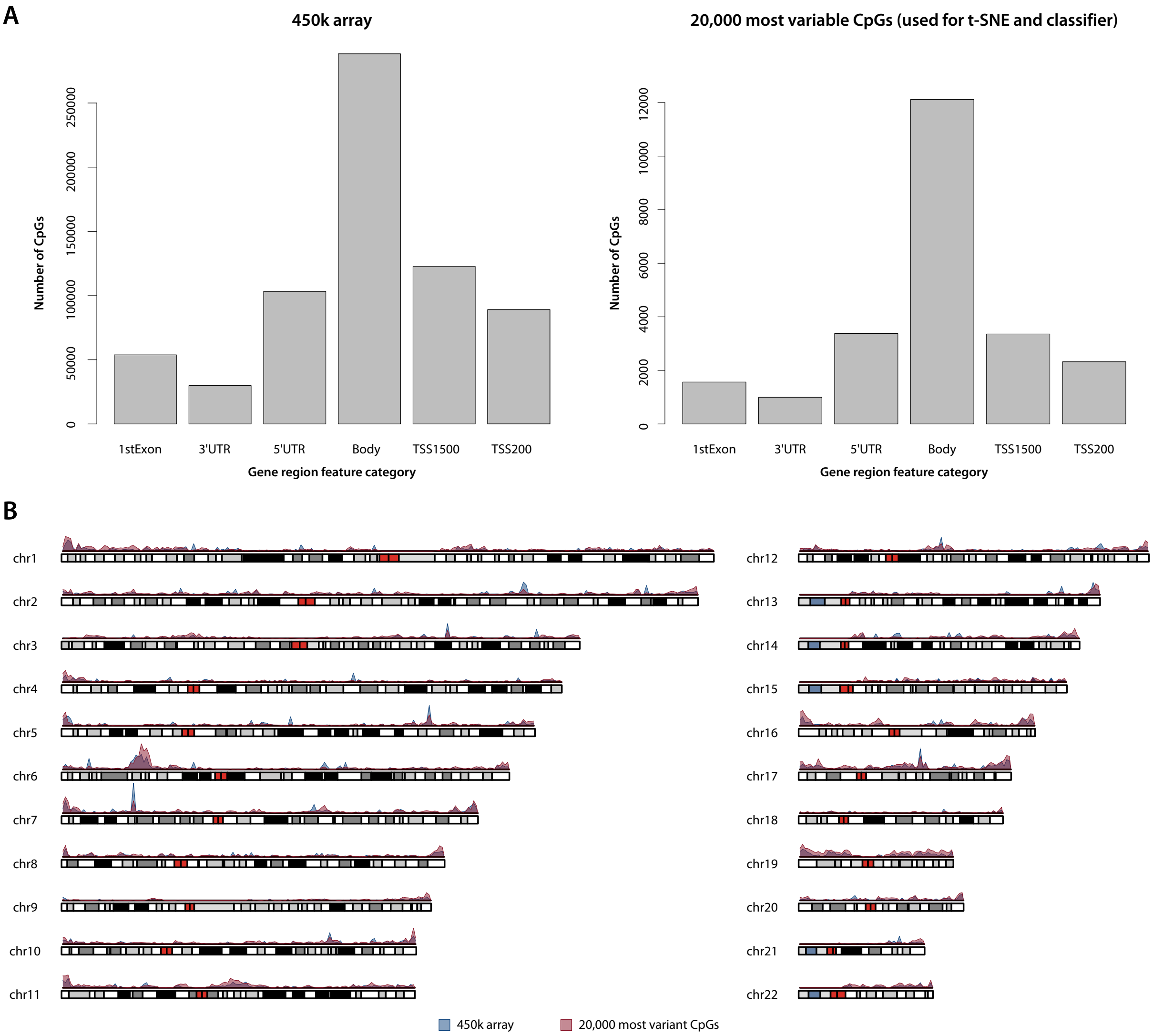

Supplementary Fig. 1 Genomic distribution of all CpG sites from the 450k array and the CpGs relevant for classification.

A Distribution of the 20,000 CpG site used by the classifier across different gene region categories compared to the 450k array design. The overall distribution is comparable and there is no enrichment in functionally relevant promotor regions.

B Karyoplot of chromosome 1 to 22 showing the density distribution per megabase of all CpGs from the 450k array design and the 20,000 most variant CpGs that were able to separate the sinonasal tumor classes. There is an overall very similar distribution without clear enrichment in specific chromosomal regions.

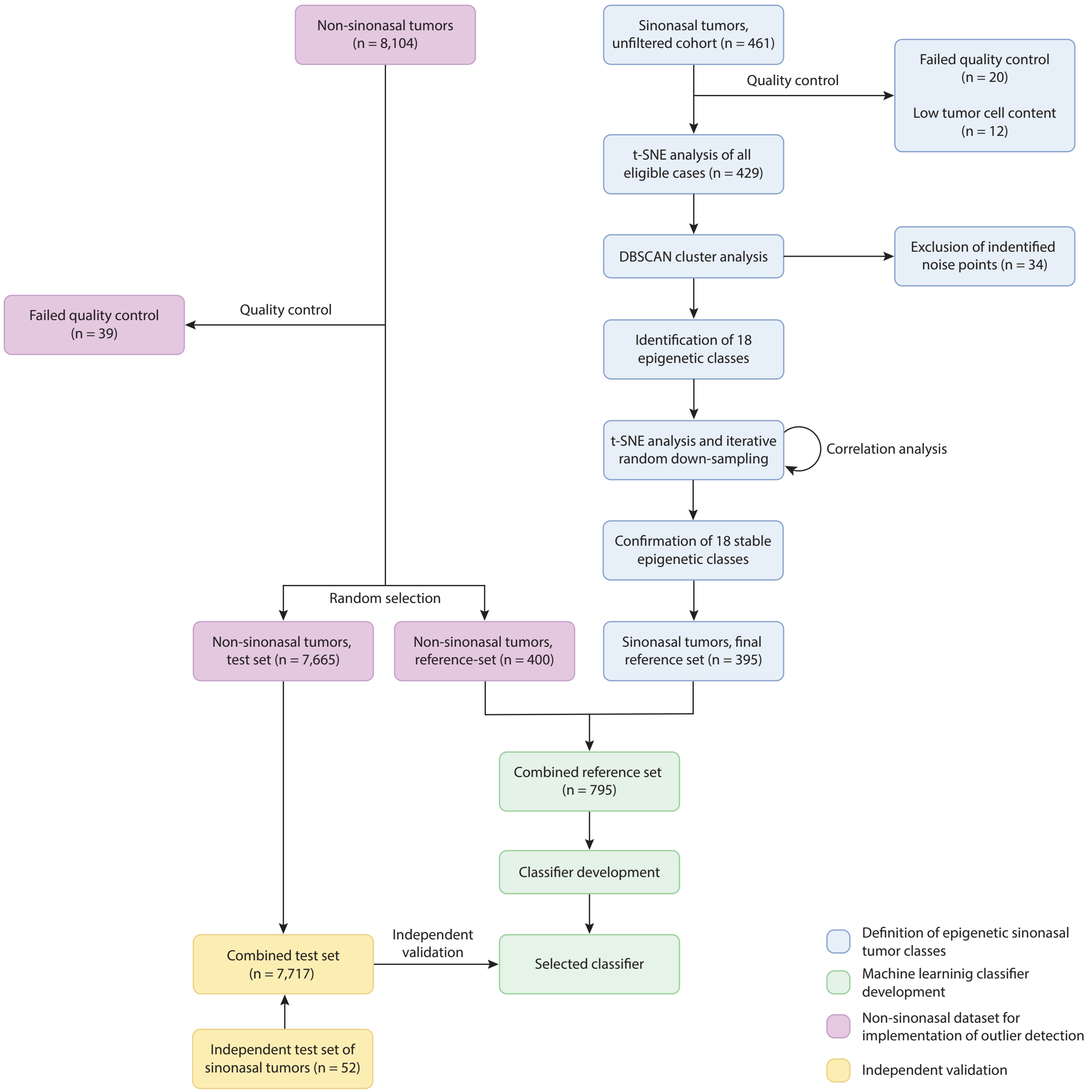

Supplementary Fig. 2 Overview of the study design. A total of 461 sinonasal tumor specimens were collected. After quality control 429 samples remained. Unsupervised clustering identified 18 epigenetic classes and revealed 34 noise data points, which were excluded from the reference cohort. Stability of the 18 classes was assessed using iterative random down-sampling and correlation analysis. Additionally, we compiled a cohort of 8,104 non-sinonasal tumor specimens to implement a supervised outlier detection. A final reference set of 395 sinonasal and 400 non-sinonasal specimens was used to train the machine learning algorithms. The resulting classifiers were validated using an independent test set of 52 sinonasal and 7,665 non-sinonasal samples.

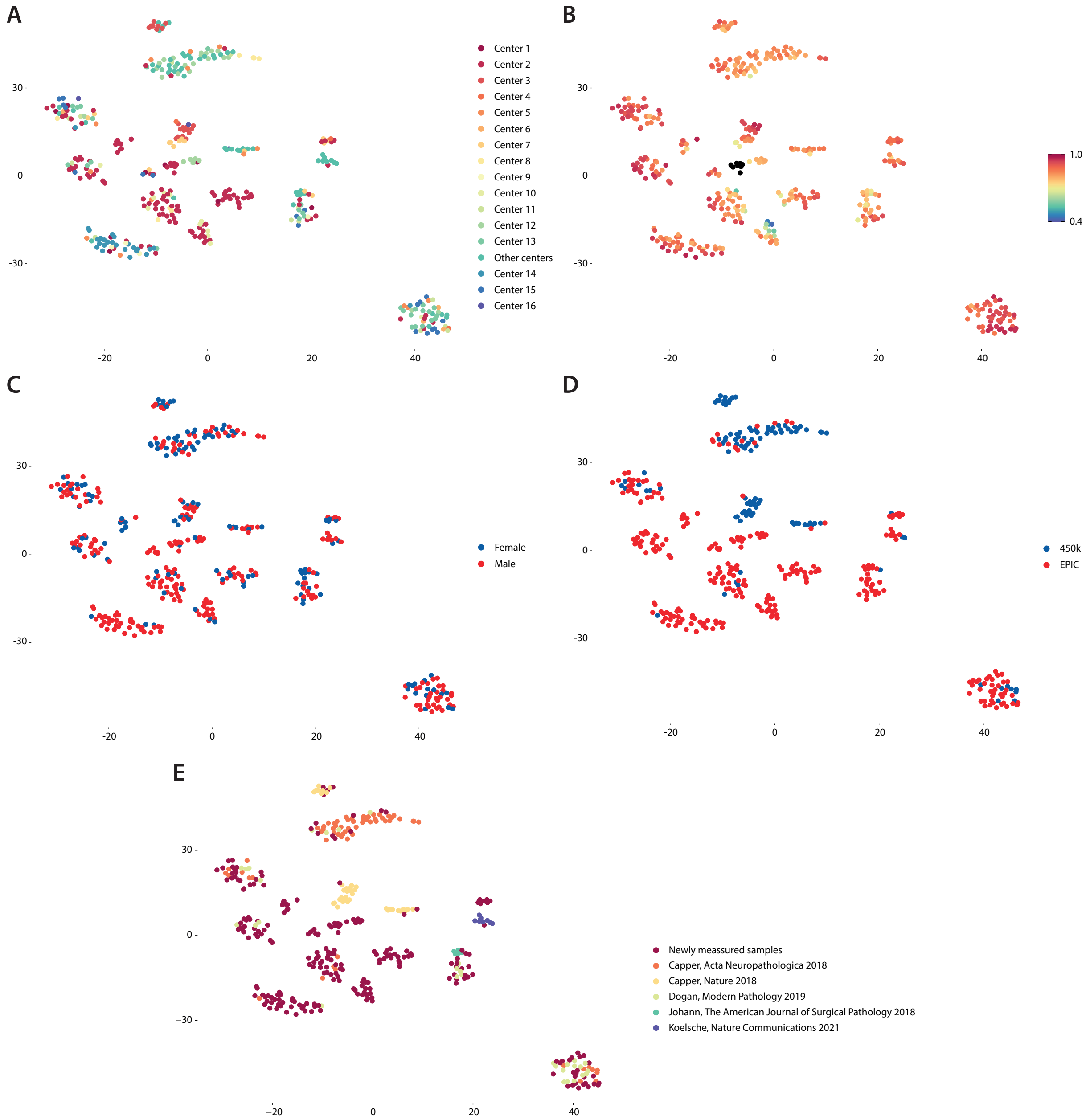

Supplementary Fig. 3 Association of potential confounding factors and stability of the different DNA methylation classes.

A Data points labeled according to the location of the site that provided the sample.

B Data points labeled according to their estimated tumor purity.

C Data points labeled according to patient sex.

D Data points labeled according to the Illumina Infinium BeadArray generation.

E Data points labeled according to study.

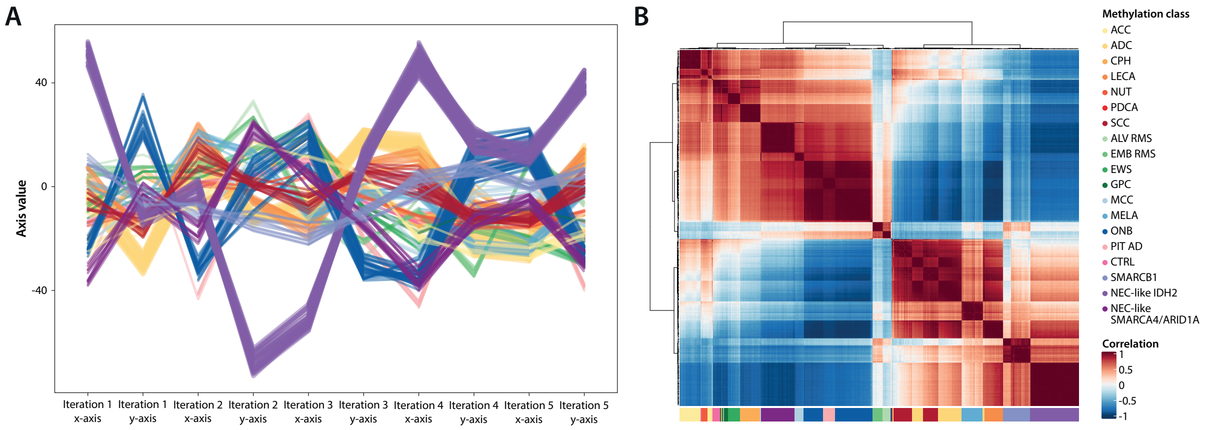

Supplementary Fig. 4 Evaluation of the stability of the identified epigenetic classes.

A Evaluation of the robustness of the different DNA methylation classes using iterative down-sampling, the plots shows the X and Y coordinates of the individual samples over the first five iterations.

B Heatmap showing the correlation for the X and Y coordinated between all samples over all 300 iterations.

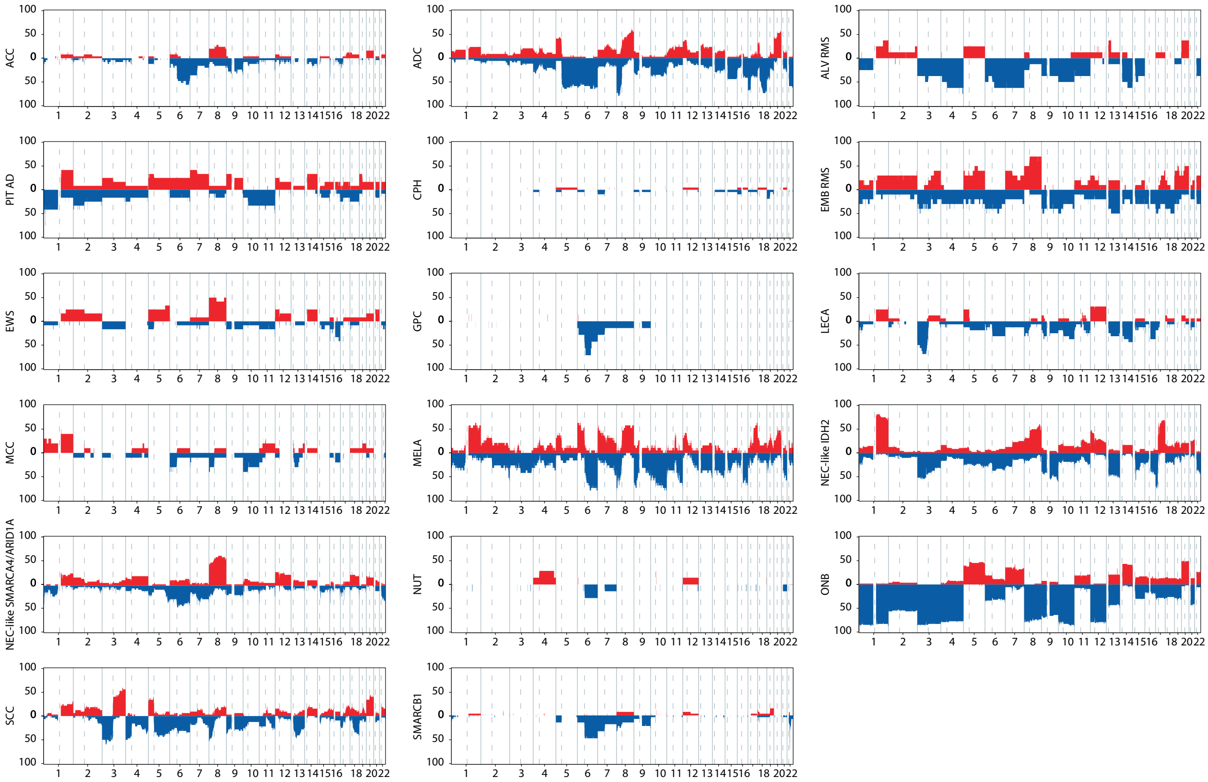

Supplementary Fig. 5 Summary copy number plots for all 17 epigenetic tumor classes.

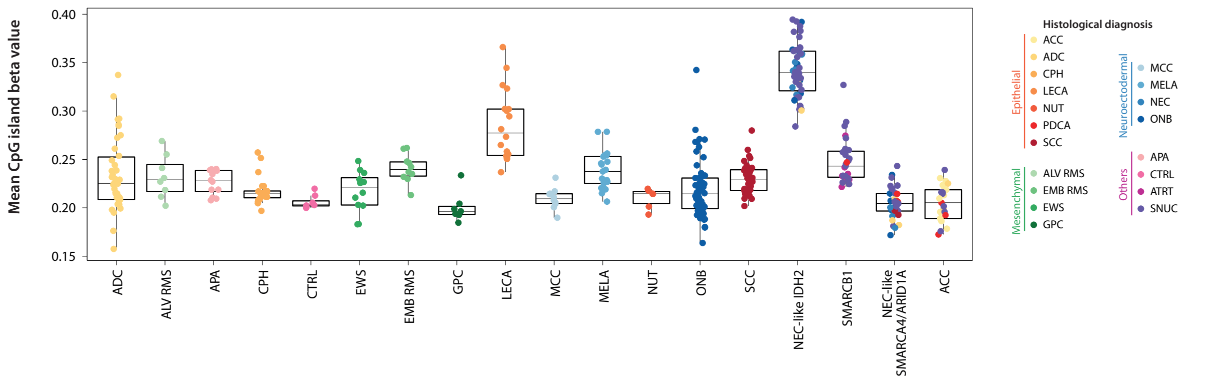

Supplementary Fig. 6 Combined box and point plot showing the mean CpG island beta value for the 17 different DNA methylation tumor classes as well as normal tissue. Upper and lower bounds of box represent the 75th and 25th percentile, respectively. The line within the box represents the median value. The upper and lower bound of the whiskers represent the largest and lowest value within 1.5 interquartile range above the 75th and below the 25th percentile, respectively. Minima and maxima are either represented by the upper and lower bound of the whiskers or as outlier points if they exceed the thresholds described above.

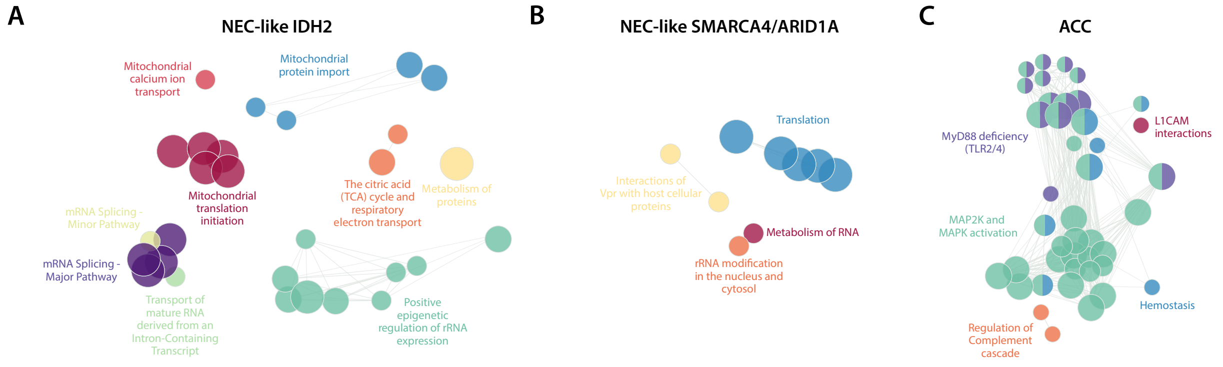

Supplementary Fig. 7 Results from functional proteomic analysis.

A Tumors from the NEC-like IDH2 class were enriched for alterations in mitochondrial processes, including citric acid cycle.

B ACC class tumors showed evidence for alterations in MAPK-related signaling pathways.

C The few significant functional terms for cases from the NEC-like SMARCA4/ARID1A class were mainly associated with translational processes.

Classifier type	Sinonasal classification		Binary (sinonasal/non-sinonasal) differentiation			
	Accuracy excluding 'unknown'	Accuracy considering second highest prediction if first prediction is 'unknown'	Sensitivity	Overall specificity	Specificity of diagnoses seen in training	Specificity of diagnoses not seen in training
Support vector machine	1.0 (47/47)	0.981 (51/52)	0.904 (47/52)	0.982 (7,524/7,665)	0.986 (6,402/6,492)	0.957 (1,122/1,173)
Random forest	0.979 (47/48)	0.962 (50/52)	0.923 (48/52)	0.969 (7,426/7,665)	0.974 (6,325/6,492)	0.939 (1,101/1,173)

Supplementary Table 1 Summary of the performance metrics of the developed machine learning classifiers.
