# Supplementary material for: DNA methylation-based classification of sinonasal tumors
Source: Nat Commun. 2022 Nov 28;13:7148. doi: 10.1038/s41467-022-34815-3 (PMC9705411; doi:10.1038/s41467-022-34815-3)
Supplement: Supplementary file 3 — Reporting Summary [file 41467_2022_34815_MOESM3_ESM.pdf]

nature portfolio

Corresponding author(s): Philipp Jurmeister

Last updated by author(s): Sep 25, 2022

Reporting Summary

Nature Portfolio wishes to improve the reproducibility of the work that we publish. This form provides structure for consistency and transparency in reporting. For further information on Nature Portfolio policies, see our Editorial Policies and the Editorial Policy Checklist.

Statistics

For all statistical analyses, confirm that the following items are present in the figure legend, table legend, main text, or Methods section.

n/a	Confirmed	
☐	☒	The exact sample size (*n*) for each experimental group/condition, given as a discrete number and unit of measurement
☐	☒	A statement on whether measurements were taken from distinct samples or whether the same sample was measured repeatedly
☐	☒	The statistical test(s) used AND whether they are one- or two-sided<br>*Only common tests should be described solely by name; describe more complex techniques in the Methods section.*
☐	☒	A description of all covariates tested
☐	☒	A description of any assumptions or corrections, such as tests of normality and adjustment for multiple comparisons
☐	☒	A full description of the statistical parameters including central tendency (e.g. means) or other basic estimates (e.g. regression coefficient) AND variation (e.g. standard deviation) or associated estimates of uncertainty (e.g. confidence intervals)
☐	☒	For null hypothesis testing, the test statistic (e.g. *F*, *t*, *r*) with confidence intervals, effect sizes, degrees of freedom and *P* value noted<br>*Give P values as exact values whenever suitable.*
☒	☐	For Bayesian analysis, information on the choice of priors and Markov chain Monte Carlo settings
☐	☒	For hierarchical and complex designs, identification of the appropriate level for tests and full reporting of outcomes
☒	☐	Estimates of effect sizes (e.g. Cohen's *d*, Pearson's *r*), indicating how they were calculated

Our web collection on statistics for biologists contains articles on many of the points above.

Software and code

Policy information about availability of computer code

Data collection	RStudio v1.3.1093, Cytoscape v3.9.1, ClueGO v2.5.9, MaxQuant v1.6.17.0, TruSight Oncology 500 v2.2 Local App Docker, Pisces v5.2.10.49, Burrows-Wheeler-Aligner v0.7.17
Data analysis	minfi v.1.38.0, wateRmelon v1.0, DMRcate v2.6.0, Rtnse v0.15, e1071 v1.7-9, randomForest v.4.6-14, glmnet 4.1-3, comunee v1.26.0<br><br>The code for application of the developed algorithm is available at https://doi.org/10.6084/m9.figshare.17144639 and also accessible via a user-friendly RShiny web interface at www.aimethylation.com

For manuscripts utilizing custom algorithms or software that are central to the research but not yet described in published literature, software must be made available to editors and reviewers. We strongly encourage code deposition in a community repository (e.g. GitHub). See the Nature Portfolio guidelines for submitting code & software for further information.

Data

Policy information about availability of data

All manuscripts must include a data availability statement. This statement should provide the following information, where applicable:

- Accession codes, unique identifiers, or web links for publicly available datasets
- A description of any restrictions on data availability
- For clinical datasets or third party data, please ensure that the statement adheres to our policy

Raw DNA methylation data of all samples that have been collected for this study have been deposited in GEO (GSE196228).
The raw proteomics and DNA sequencing data are protected and are not available due to data privacy laws. The processed proteomics data are available at FigShare (https://doi.org/10.6084/m9.figshare.17144639). DNA sequencing data will only be shared upon reasonable request and only for non-commercial purposes due to privacy restrictions. Requests should be directed to the corresponding author and will be processed within 14 business days.
For part of the study, publicly available data was retrieved from the TCGA database (https://www.cancer.gov/tcga).

Human research participants

Policy information about studies involving human research participants and Sex and Gender in Research.

Reporting on sex and gender	Only information on sex was collected for this study. Information is provided in Supplementary Table 3 and 4, a summary is given in the 'Population characteristics' field below. Gender was not collected for this study.
Population characteristics	Adenoid cystic carcinoma; median age/range: 61,5 (41 - 75) years; male:female ratio: 16:9 (1.8)<br>Adenocarcinoma; median age/range: 63 (45 - 82) years; male:female ratio: 33:3 (11.6)<br>Alveolar rhabdomyosarcoma; median age/range: 30 (5 - 74) years; male:female ratio: 4:4 (1)<br>Craniopharyngioma; median age/range: 48,5 (3 - 69) years; male:female ratio: 11:10 (1.1)<br>Control tissue; median age/range: NA years; male:female ratio: 7:1 (7)<br>Embryonal rhabdomyosarcoma; median age/range: NA years; male:female ratio: 7:3 (2.3)<br>Ewing's sarcomas; median age/range: 16,5 (8 - 28) years; male:female ratio: 7:5 (1.4)<br>Lymphoepithelial carcinoma; median age/range: 46 (35 - 71) years; male:female ratio: 15:3 (5)<br>Merkel-cell carcinoma (neuroendocrine carcinoma of the skin); median age/range: 77,5 (61 - 97) years; male:female ratio: 4:6 (0.7)<br>Mucosal melanoma; median age/range: 70 (43 - 90) years; male:female ratio: 13:8 (1.6)<br>Neuroendocrine carcinoma-like, IDH2 mutant; median age/range: 50 (30 - 87) years; male:female ratio: 33:15 (2.2)<br>Neuroendocrine carcinoma-like, SMARCA4/ARID1A enriched; median age/range: 58 (15 - 91) years; male:female ratio: 21:12 (1.75)<br>NUT midline carcinoma; median age/range: 29 (29 - 29) years; male:female ratio: 6:0 (-)<br>Olfactory neuroblastoma; median age/range: 59 (20 - 82) years; male:female ratio: 26:31 (0.8)<br>Pituitary adenoma; median age/range: 47 (28 - 61) years; male:female ratio: 5:7 (0.71)<br>Sinonasal glomangiopericytoma; median age/range: 65 (39 - 94) years; male:female ratio: 6:1 (6)<br>Squamous cell carcinoma; median age/range: 67 (35 - 87) years; male:female ratio: 28:6 (4.6)<br>Sinonasal carcinoma, SMARCBl altered; median age/range: 53 (20 - 81) years; male:female ratio: 13:14 (0.93)<br>Sinonasal glomangiopericytoma; median age/range: 65 (39 - 94) years; male:female ratio: 6:1 (6)<br>Squamous cell carcinoma; median age/range: 67 (35 - 87) years; male:female ratio: 28:6 (4.6)<br>Sinonasal carcinoma, SMARCBl altered; median age/range: 53 (20 - 81) years; male:female ratio: 13:14 (0.93)
Recruitment	Suitable samples were identified in retrospective using the laboratory information system of the respective institutes of pathology.
Ethics oversight	Ethics approval was granted by the ethics committe of the Charite - Universitätsmedizin Berlin, Chariteplatz 1, 10117 Berlin, Germany

Note that full information on the approval of the study protocol must also be provided in the manuscript.

Field-specific reporting

Please select the one below that is the best fit for your research. If you are not sure, read the appropriate sections before making your selection.

[x] Life sciences [ ] Behavioural & social sciences [ ] Ecological, evolutionary & environmental sciences

For a reference copy of the document with all sections, see nature.com/documents/nr-reporting-summary-flat.pdf

Life sciences study design

All studies must disclose on these points even when the disclosure is negative.

Sample size	Minimum sample size per group was set at 6, similarly to previously published methods (e.g. Capper et al. 2018).
Data exclusions	Samples were selected based on the following predefined critea:<br>(1) DNA methylation analysis<br>- Samples with a mean detection p-value >0.05 for >5% of all valid CpGs were excluded<br>- Samples with unexpectedly flat copy number profiles (indicating low tumor cell content) were excluded<br>- Samples that were identified as noise/singularity points using the HDBSCAN duster algorithm (indicating divergent DNA methylation profiles) were excluded<br>(2) Proteomics<br>- Samples with <1000 identified proteins were excluded
Replication	The stability of the DNA methylation classses was confirmed using iterative random downsampling as previously described (Capper et al. 2019, Koelsche et al. 2021). High correlation scores proofed the stability of the defined classes. Five repetitions of the classifier development and evaluation led to similar results as with our final classifier, confirming the stability of the procedure.
Randomization	Samples for the sinonasal test set were acquired seperately after classifier development using the reference set, so randomization was not necessary. For the non-sinonasal cohort, 5% of the samples of each category from the non-sinonasal cohort (n = 400) were randomly selected for the training cohort, the remaining samples were assigned to the reference cohort.
Blinding	The unsupervised methods that were used to identify different DNA methylation classes were blinded to the conventional histopathological. diagnosis.

Reporting for specific materials, systems and methods

We require information from authors about some types of materials, experimental systems and methods used in many studies. Here, indicate whether each material, system or method listed is relevant to your study. If you are not sure if a list item applies to your research, read the appropriate section before selecting a response.

Materials & experimental systems

n/a	Involved in the study	
☐	☒	Antibodies
☒	☐	Eukaryotic cell lines
☒	☐	Palaeontology and archaeology
☒	☐	Animals and other organisms
☒	☐	Clinical data
☒	☐	Dual use research of concern

Methods

n/a	Involved in the study	
☒	☐	ChIP-seq
☒	☐	Flow cytometry
☒	☐	MRI-based neuroimaging

Antibodies

Antibodies used	UCHL1 (abcam, 13C4, 1:1000, catalog number ab8189), KRT18 (BioGenex, clone DC-10, dilution 1:1000, catalog number AM143-5M)
Validation	Antibodies were validated using adequate positive and negative controls (human neural tissue for UCHL1 and human cancer tissue for KRT18).
