# Supplementary material for: DNA methylation-based classification of sinonasal tumors
Source: Nat Commun. 2022 Nov 28;13:7148. doi: 10.1038/s41467-022-34815-3 (PMC9705411; doi:10.1038/s41467-022-34815-3)
Supplement: Supplementary file 4 — Description of Additional Supplementary Files [file 41467_2022_34815_MOESM4_ESM.pdf]

Description of Additional Supplementary Files

File Name: Supplementary Data 1
Description: Description of the identified molecular sinonasal tumor classes based on DNA methylation data.

File Name: Supplementary Data 2
Description: Metadata of all cases from the sinonasal reference set.

File Name: Supplementary Data 3
Description: Metadata of all cases from the sinonasal test set.

File Name: Supplementary Data 4
Description: List of all cases from the non-sinonasal set.
